# Supplementary material for: Pregnant women’s migration patterns before childbirth after large-scale earthquakes and the added impact of concerns regarding radiation exposure in Fukushima and five prefectures
Source: PLoS One. 2022 Aug 1;17(8):e0272285. doi: 10.1371/journal.pone.0272285 (PMC9342739; doi:10.1371/journal.pone.0272285)
Supplement: Attachment — Submitted filename: Response to Reviewers.docx [file pone.0272285.s001.docx]

Dear Dr. Gayle E. Woloschak,

Thank you for allowing me to submit a revised draft of my manuscript titled “Pregnant women’s migration patterns before childbirth after a large-scale earthquake and the added impact of concerns regarding radiation exposure in Fukushima and five prefectures” to *PLOS ONE*. We appreciate the time and effort that you and the reviewers have dedicated to providing your valuable feedback on our manuscript. We are grateful to the reviewers for their insightful comments on our paper. We have been able to incorporate changes to reflect most of the suggestions provided by the reviewers. We have highlighted the changes within the manuscript. Here is a point-by-point response to the reviewers’ comments and concerns.

Comments from Reviewer 1

- Comment 1: *In higher flow rates of pregnant women in Fukushima prefecture, authors referred "...concerns about radiation exposure have had long-term effects on women’s migration patterns.". How do authors manage the data of evacuees from the 13 evacuation ordered areas by the national government who needed to move out from their home town?*

Response: Thank you for pointing this out. We agree that it is important to explain how to manage the evacuation ordered areas. The background of pregnant women's migration varies: some pregnant women moved because of concerns about the effects of radiation, some pregnant women moved due to physical damage to their housing environment or medical building collapses, and some pregnant women moved because of government restrictions for returning. We have explained that calculating the rate of flow of pregnant women determines how to manage evacuation ordered areas. We have added the following sentences to explain this point at page 10, lines 151-158.

“It is possible that the areas with government restrictions for returning are not areas where pregnant women are willing to move, but areas where they are ordered to move by the government, and the background that determined their movement is different. However, the Vital Statistics birth registry used in this study could not grasp these backgrounds. Therefore, in this study, the 13 areas ordered for evacuation were also treated as residential areas of pregnant women, without distinguishing between the intention of pregnant women and administrative restrictions on return.”

- Comment 2: *Are there any difference in outflow rate and inflow rate between evacuation area and non-evacuation area? It is very important to consider the effect of radiation concerns.*

Response: Yes, there were. However, the change procedure of residential address after the disaster was not mandatory, and there are still some disaster victims who left their residential address in the evacuation area. Therefore, we decided that we could not treat the investigation by the municipality as an accurate result, and we reported the migration by prefecture in this study. We have added this information to the “Study strengths and limitations” sections on Page 21, lines 347-354.

"The Fukushima Health Management Survey conducted by the Fukushima Prefecture follows up on the pregnant women living in the affected areas in the municipal units, including the 13 evacuation ordered areas. In the future, we intend to use these data to conduct a detailed survey on a municipal unit. Furthermore, it is necessary to clarify whether there are differences in the pregnant women outflow and inflow rates by governmental restrictions on returning within the affected prefecture through a more detailed municipality analysis.”

- Comment 3: *Authors need to correct the description in Reference.*

Response: Thank you for pointing this out. We have revised and have incorporated your suggestion throughout the reference section. We have added three new references in our revised manuscript. In addition, all the in-text citation numbers were appropriately updated.

Comments from Reviewer 2

- Comment 1: *I wonder, what will you recommend to health clusters or governments as a notion from your research in the conclusion. Mothers did not and cannot return to their original residential place not only because of fear of the radiation, but governmental restrictions for returning. There could be no conclusive scientific evaluation on which is better to go back or not. I appreciate it if you could add some notion from your discussion in conclusion on the preparedness of affected and non-affected prefectures and health clusters.*

Response: Thank you for this suggestion. We agree that it is important to discuss the preparedness of the prefectures and health clusters. We have added the following sentences to explain this point on Page 20, lines 316-324.

“Therefore, as a preparation for large-scale disasters in the non-affected area, during the acute phase of a disaster, the number of pregnant women who have been evacuated from the affected area may increase, causing a temporary congestion in perinatal care. Hence, health clusters or governments need to establish a medical system or find methods to grasp the information regarding the prenatal checkups’ process that can appropriately disperse evacuated pregnant women. Moreover, preparation for a major disaster in the affected area should include establishing a plan for receiving assistance during the disaster and means to sequentially publicize the results of a multifaceted investigation of restrictions for return.”

- Comment 2: *Because you are dealing with various earthquakes, use "large-scale earthquakes". Same with the short title. Use "disasters".*

Response: Thank you for pointing this out. We agree with this and have incorporated your suggestion.

- Comment 3: *The meaning of "utilities" is not clear. I recommend to use "lifelines" instead.*

Response: Thank you for this suggestion. Using a dictionary, we found that public systems, such as electricity, gas, and water generally use utilities rather than lifeline. We have revised this appropriately for clarity. The revised text on Page 3, line 48 now reads as “utilities, like electricity, gas, water”.

- Comment 4: *Ref. 12 and 13 do not seem to be media reports. Add brief explanation of the notions of References 12 and 13, how the already complex radiation disaster will result in a "complex disaster"*

Response: Thank you for pointing this out. We agree with this comment. Therefore, we have quoted the following sentence on Page 4, lines 62-65 from References 12 and 13.

“The mechanism of health effects caused by the radiation has not yet been clarified, and opinions are divided among experts, which possibly cause anxiety not only among residents of the affected areas but also among the medical members who support them, and may even lead to harmful rumors about the affected areas.”

- Comment 5: *I think it is better to explain how responders and governments created "emergency consultation centers for radiation exposure for pregnant women" after GEJE including their locations, duration, and the roles before citing Ref. 14. Ref. 15 is a paper on environmental exposure, but not much oriented toward radiation. Add the notion from Ref. 15.*

Response: Thank you for pointing this out. We agree that it is important to explain the details of "emergency consultation centers for radiation exposure for pregnant women." Therefore, we have quoted the details from the Fukushima Prefecture’s website. Then, for environment exposure, we quoted the effects of environmental exposure on pregnant women from Reference 18, 19 and20. This information was added to Introduction on Page 4-5, lines 65-79.

- Comment 6: *It seems Ref. 18 should be in place of Ref. 19. Reorganize the references and citations.*

Response: Thank you for this suggestion. We agree with this and have incorporated your suggestion. We have added three new references in the revised version of our manuscript. In addition, all in-text reference numbers were corrected and updated appropriately.

- Comment 7: *Are you sure [15] is correct, not [14]?*

Response: Thank you for pointing this out. We agree with this and have incorporated your suggestion.

- Comment 8: *It is better to use "affected area" instead of "disaster area"*

Response: Thank you for pointing this out. We agree with this and have incorporated your suggestion.

- Comment 9*: If the data is publicly available, indicate the URL or reference for the birth registry. Indicate where the readers can find the data on the mothers’ migration. If you specifically requested the data from Ministry, describe so as written in Line 115.*

Response: We agree with this and have incorporated your suggestion. We have added the sentence that we have requested the data from the Ministry on Page 5 lines 85-87.

- Comment 10*: Use "earthquakes"*

Response: Thank you for pointing this out. We agree with this and have incorporated your suggestion.

- Comment 11: *If you are explaining the purpose of your study, "aims to clarify" instead of "suggests" may suit.*

Response: Thank you for pointing this out. We agree with this and have incorporated your suggestion.

- Comment 12: *Please mention that the seismic resistance of houses and buildings were different by time. The experience of 1995 GHAE made the anti-seismic reinforcement in many other areas in Japan. Thus, the same seismic intensity does not destroy the same number of houses and buildings in the target earthquakes.*

Response: Thank you for pointing this out. We agree with this comment. Therefore, we added the sentence explaining that after disasters, the Building Standard Law was revised and the seismic strength of houses was improved. This information was added to Methods on Page 7, lines 110-113.

“Furthermore, Japan is prone to disasters such as typhoons and earthquakes and the Building Standard Law has been revised many times, each time increasing the strength of buildings. Therefore, the seismic resistance of houses and buildings were different over time.”

- Comment 13: *The epicenter was far out in the Pacific Ocean in GEJE. "severely affected prefecture" is fine.*

Response: Thank you for pointing this out. We agree with this and have incorporated your suggestion.

- Comment 14: *I think the denominator is the number of births in the relevant prefecture which is the same as the denominator of outflow. "the number of births for which the place of residence was not in the relevant prefecture" can be very large. Confirm the correctness.*

Response: Thank you for pointing this out. The denominator is the number of residents in one prefecture, since the outflow is the rate of pregnant women whose place of residence is in the affected prefecture and whose place of notification is in the 46 prefectures except for their place of residence. For the other, the denominator is the number of residents in 46 prefectures except for the affected prefecture, since the inflow is the rate of pregnant women whose place of notification is the affected prefecture among those whose place of residence is not the affected prefecture. Therefore, the denominator information size is different for outgoing and incoming pregnant women. We have corrected it to prevent any misunderstanding by readers on Page 9-10, lines 142-151

- Comment 15: "life lines"?

Response: Thank you for this suggestion. Using a dictionary, we found that public systems, such as electricity, gas, and water generally use utilities rather than lifeline. We have revised that it to “utilities, such as electricity, gas, and water” for clarity on Page 17, line 250.

- Comment 16: *Is Ref. 15 correct?*

Response: Thank you for pointing this out. The content refers to what happened after the GEJE, and we have decided that Reference 15 is not relevant and therefore, removed it from the reference list.

- Comment 17*: # Ref. 18: "Kobayashi Y" instead of "Yukiko K".*

Response: Thank you for pointing this out. We revised with this and have incorporated your suggestion.
