# Supplementary material for: Pregnant women’s migration patterns before childbirth after large-scale earthquakes and the added impact of concerns regarding radiation exposure in Fukushima and five prefectures
Source: PLoS One. 2022 Aug 1;17(8):e0272285. doi: 10.1371/journal.pone.0272285 (PMC9342739; doi:10.1371/journal.pone.0272285)
Supplement: Attachment — Submitted filename: Response_to_Reviewers.docx [file pone.0272285.s002.docx]

Dear Dr. Gayle E. Woloschak,

Thank you for allowing us to submit a revised draft of our manuscript, titled “Pregnant women’s migration patterns before childbirth after a large-scale earthquake and the added impact of concerns regarding radiation exposure in Fukushima and five prefectures” to *PLOS ONE*. We highly appreciate your and the reviewers’ time and effort dedicated toward our manuscript as well as your valuable feedback. We are grateful to the reviewers for their insightful comments on our paper. We have incorporated the changes needed to reflect most of the suggestions provided by the reviewers. Additionally, we have highlighted the changes within the manuscript. Here is a point-by-point response to the reviewers’ comments and concerns.

Comments from Reviewer 1

- Comment 1: *Reviewer #1: Comment 1. Please correct the authors name.*

Response: Thank you for pointing this out. We have revised the names appropriately.

- Comment 2: *Is there really available of Ref. 43 (Ryoko F. ) ? The reviewer can't find the reference.*

Response: Yes, there is. However, it can only be confirmed on print media. This literature is written in Japanese and the author's name is Furukawa R. It has been corrected. Additionally, we have included a source link for the ease of reference. Please refer to the following URL:

<https://researchmap.jp/read0112116/misc/37229744>

Moreover, we have added Japanese title and the URL to the “Reference” sections on Page 27, lines 488-491.

“Furukawa R. [Current reality about satogaeri bunben to support pregnant couples’ choice of the birth place]. Syussan basyo no sentaku wo enjo suru tame no satogaeri bunben no genjou (in Japanese) [in print] Japan Society of Matern Health. 2018;59(2): 560-568. (<https://researchmap.jp/read0112116/misc/37229744>)”

Comments from Reviewer 2

- Comment 1: *# Line 44: The Abstract section needs conclusive statement. Add what is necessary to be a resilient society summarizing the notion from Lines 319-324.*

Response: Thank you for this valuable suggestion. We have added the following sentences to the abstract on page 3, lines 40-43. With the addition to the abstract, it has been revised overall and is well with the journal’s stipulated limit (300 words)

“These results suggested that plans for receiving assistance and support that considers the peculiarities of disaster related damage and pregnant women’s migration patterns are needed in both the affected and non-affected areas.”

- Comment 2: *# Line 79: "fetal" or "fetuses"?*

Response: Thank you for pointing this out. We revised this appropriately.

- Comment 3: *# Line 87: "applied to the Ministry for Birth registry data use" or "obtained the Birth registry data from the Ministry" may fit.*

Response: Thank you for this valuable suggestion. We have revised this appropriately for clarity by incorporating your suggestion.

- Comment 4: *# Line 139: "that was close to the epicenter" is the correct expression.*

Response: Thank you for pointing this out. We have revised this appropriately using your suggestion.

- Comment 5: *# Line 248: Insert "Niigata Chuetsu earthquake, " between GHAE and GEJE.*

Response: Thank you for this valuable suggestion. We revised with this and have incorporated your suggestion.

- Comment 6: *# Line 260: Is "expectant pregnant women" correct? Not "expectant mothers"?*

Response: Thank you for pointing this out. We have revised this term for clarity.

- Comment 7: *# Line 364: I think it is better to add a notion from LInes 319-324 as well as in the abstract.*

Response: Thank you for this suggestion. We have added the following sentences on Page 22, lines 364-366,

“Therefore, it is necessary to establish plans for assistance and support that consider the damage caused by disasters and migration of pregnant women after disasters in both the affected and non-affected areas.”
